# Linguistic expressions of negative stances: A conversation analysis of turn-medial particle *dai* in Jishou dialect (Hunan Province, China)

**DOI:** 10.3389/fpsyg.2023.1018648

**Published:** 2023-02-21

**Authors:** Feng Liu, Xi Li, Rurong Liu, Jianyu Zeng

**Affiliations:** ^1^College of Foreign Languages, Jishou University, Jishou, Hunan, China; ^2^School of Foreign Languages, Hunan University of Finance and Economics, Changsha, Hunan, China; ^3^College of English, Xi'an International Studies University, Xi'an, Shaanxi, China

**Keywords:** Jishou dialect, turn-medial particle *dai*, marker of negative affective stance, conversation analysis, interactional (socio) linguistics

## Abstract

The study focuses on the syntactic functions and prosodic features of the turn-media particle *dai* in Jishou dialect, Hunan Province, China, as well as its distributions and interactional functions across eight different contexts. The research utilizing a corpus of 70 h consisting of 300,000 characters of the Jishou dialect, employed the conversation analysis (CA) method to analyze the interactional behaviors of *dai*. The results show that *dai* serves as an overt marker of speakers' negative stances, including complaining and criticizing. It is treated as an emerging product continuously shaped by diverse factors, such as context, sequential positioning, prosodic manifestation in talk-in-interaction, and its influence on the subsequent development of the conversation.

## 1. Introduction: Particles and language

Particles are ubiquitous in languages across the world. They have been cross-linguistically discussed in terms of their function of marking speakers' stance, e.g., *oh* in English (Schiffrin, 1987; Heritage, 1998); *yo, ne, sa, zo*, and *no* in Japanese (Cook, 1990, 1992; Maynard, 1993, 2002; Tanaka, 1999), and *kwun, ney, tela*, and *ta* in Korean (Lee, 1993; Strauss, 2005; Strauss and Ahn, 2007). These particles exist within a wide variety of interactional contexts and serve to express surprise, empathy, and degrees of certainty. Examples of these include “*a, ou, ya, ba, ne”* in Mandarin (Li and Thompson, 1981; Li et al., 1982; Wu, 1997, 2004), “*la, lo, wo*” in Cantonese (Luke, 1990; Matthews and Yip, 2013), “*nâ, nia*” in Thai (Horie and Iwasaki, 1996), and “*nii(n), joo, kato*” in Finnish (Hakulinen and Seppänen, 1992; Sorjonen, 1997, 2001).

Given the pervasiveness of particles within and across languages and their discourse-pragmatic functions, their research can provide keen insights into the socio-contextual elements that underlie various aspects of speakers' stances in relation to the participants, the theme of the talk, the information presented, and so on.

In the article, conversation analysis (CA) is employed to investigate the interactional functions of one such turn-medial particle,^1^*dai*, in the Jishou dialect of Western Hunan Province, China. We chose ‘*dai'* as the focal point of our study for two reasons: first, it plays a crucial role in indicating the speaker's emotional stance, and second, it is unique to the Jishou dialect and is not present in other dialects spoken in the Hunan Province, as confirmed by both literature and field research. We identified and tracked its usage within a corpus of audio-taped interactions in which native speakers of Jishou interact face-to-face with numerous interlocutors in various contexts. The study illustrates that *dai* serves as a clear indicator of the speaker's negative stance, and it is utilized by speakers to express complaints and criticisms within negative interactional contexts,^2^ as is presented in excerpt 1 below:

(1) (Households)

(In this scenario, person M^3^ believed that person R had already reached his destination, Jishou City. M then expressed her complaint to R over the phone, stating that the train was late. However, it was later revealed that R had only just arrived in Huaihua City, which is a two-hour journey away from Jishou City).

1M:  *aiyo*,                       *cai   dao         huaihua      a*.
        (exclamation),        just  arriving   in (city)      PRT
        Uh-oh, (you are)    just  arriving   in *Huaihua*.
2M:   → *wo*  ***dai***:    *hai           yiwei      ni     zao      dao         le.*
                     I PRT still          thought  you   early   arrive      PRV
                     I DAI thought    that        you   had     arrived    early.
                                                                                  (in Jishou)
3M: *  aiyo,   na    ni    xiaci     bie   zai     zuo      huoche        le.*
*         (exclamation), then  you  next time N  again take train CRS*
         Ah, then you (had better) not take the train next time.

Excerpt 1 illustrates when the *dai* turn-at-talk is used to address speaker M's complaints (line 2). Her negative stance can also be seen in her suggestion that R had better not take the train next time (line 3). Prosodically and lexically, the prolonged particle *dai* and two prefaced exclamations *aiyo* mainly contribute to achieving a negative affective stance. In the article, we will demonstrate that turn-medial particle *dai* is an overt marker of an emergent negative stance in talk, showing participants' orientation to complaints and criticisms.

## 2. Previous research on *dai*

The Jishou dialect is spoken in an area at the border between the *Xiang* dialect, which is spoken in Hunan Province, China, and Southwestern Mandarin. It displays characteristics of both dialects, combining features of the *Xiang* dialect and Southwestern Mandarin in its phonetic system. For instance, when ancient-voiced initials are pronounced as plosives or affricatives, their tones are transformed into *Ping-sheng* (level ones) in the same unaspirated way as the *Xiang* dialect does. However, there is no *Ru-sheng* (entry tone) in the Jishou dialect. Under this condition, ancient *Ru-sheng* characters are pronounced in a rising tone whose tone pitch is similar to the usual tone pitch of Southwestern Mandarin. Morpho-syntactically, abundant retroflexed er-suffixed (-儿) words, reduplicative constructions, and shared syntax ingredients with Southwestern Mandarin in the Jishou dialect demonstrate that the Jishou dialect is far closer to Southwestern Mandarin than the *Xiang* dialect (Xiang, 2011). In addition, the fact that ethnic minorities account for 70% of the total population in Jishou city contributes to long-term contact among the *Han, Tujia*, and *Miao* populations, with distinctive features of the Jishou dialect induced by regional language contact (Li, 2002; Xiang, 2009).

Typical for the Jishou dialect, the particle *dai* is common in ordinary conversations. Previous studies on *dai* (e.g., Li, 2002; Xu, 2006; Yang, 2008) argued morphologically that it serves as an infix in the verb reduplication “V *dai* V” and adjective reduplication “A *dai* A.” However, in the former sense, it is placed at the sentence's predicate, as is presented in excerpts 2 and 3. In the latter sense, it acts as the predicate or complement, as is presented in excerpts 4 and 5. Besides, they also point out that the embeddedness of the particle *dai* adds speakers' subjective stances to their utterances. Thus, “V *dai* V” and “A *dai* A,” compared with those without *dai*, present speakers' stronger deontic modality (Excerpts 2–5 are quoted from Li, 2002: p. 250–258).

(2)    *wo    naoke   yun **        dai**           yun*.
         my   head     faint       PRT         faint
         I       am        feeling   strongly   faint.
(3)     *hu               li          de       shui     kai **     dai**         kai.*
         kettle          inside   PRT    water   boil     PRT       boil
         The water   inside   the       kettle   keeps boiling   violently.
(4)     *na   tiao   po     gao   **dai**     gao,   pa            si          ge       ren.*
         that  C      slope high  PRT   high, climb       exhaust C        people
         That slope is      so      high and     exhausting to        climb up.
(5) *    ta     zuo shi      guoxi               dai       guoxi.*
         3SG do   things careful            PRT     careful
         He    is    quite   conscientious about his work.

Unlike previous research, this study identified 54 instances where *dai* functions as an infix in verb and adjective reduplication, accounting for only 2% of total occurrences of *dai*. The remaining 98% function as turn-medial particles, demonstrating that *dai* plays an important role in interactions among speakers of Jishou, as is presented in excerpt 1.

Given the claims that the turn-medial particle *dai* is highly emotionally charged and frequently used in interactions, this study, which is based on a corpus of 70 h comprising approximately 300,000 characters of the Jishou dialect, employs CA to explore its interactional functions. This study also seeks to gain a deeper understanding of the functional motivations behind its use, not just through the analysis of specific linguistic features but also by examining the actions and attitudes that it accomplishes in the course of the interaction, as evidenced by the participants' conduct.

The remainder of this article is organized as follows. “Section 3” introduces the analytical and methodological framework and provides an overview of the data analyzed, the transcription procedures, and the contextual distribution of *dai*. “Sections 4 and 5” demonstrate *dai's* syntactic functions, prosodic features, and sequential functions. “Section 6” presents findings regarding the interactional functions of *dai* in the initiative and responsive positions. The final section summarizes the findings presented in the main sections and discusses the implications of this study.

## 3. Methodology and data

### 3.1. The function of particles in social interaction

CA is a field of research where a conversation is viewed as a primordial site for and a basic form of social interaction, which is this study's major theoretical and methodological framework. Social interaction is structurally organized by participants and is “a describable domain of interactional activity exhibiting stable, orderly properties that are the specific and analyzable achievements of speakers and hearers” (Zimmerman, 1988: 407). The main objective of CA is to examine and describe the structural elements of everyday conversations and other forms of talk-in-interaction that enable people to establish a shared understanding of the interaction.

From a CA perspective, one key aspect that underlies the organization of social interaction is “recipient design,” i.e., “the multitude of respects in which the talk by a party in a conversation is constructed or designed in ways that display an orientation and sensitivity to the particular other(s) who are the co-participants” (Sacks et al., 1974: p. 727). In the organization of their action, participants ordinarily consider the contingencies as they demonstrate in their next moves what sense they make of a prior speaker's action. Accordingly, to understand the participants' “then-relevant” sense of these contingencies, the immediately subsequent moves by participants commonly become analytic loci for CA. In short, this study mainly uses subsequent talk as a “proof procedure” in the analysis.

From the perspective of CA, the interactional functions of the turn-medial particle *dai* can be accomplished interactionally through the use of linguistic resources or other practices. Thus, the interactional functions of *dai* are treated as an emerging product that is shaped by and shapes the subsequent development of interaction. In this process, linguistic resources or other practices play a constitutive role in contributing to the functions accomplished by the particle *dai*. The interactional functions of particle *dai* are mutually dependent, thereby “maintaining or altering the sense of the activities and unfolding circumstances in which they occur” (Heritage, 1984: p. 140). Our study outlines three major areas for studying the particle's functions: (a) context; (b) sequential positioning; and (c) prosodic manifestation. Our study sheds light on how linguistic resources play a role in achieving social action and mutual understanding, and it can be seen as a contribution to the expanding field of research on Chinese dialects within interactional linguistics.

### 3.2. The data^4^ and transcription conventions

Our study's corpus consists of telephone and face-to-face conversations among family members, friends, or acquaintances. The authors collected these conversations mainly in eight contexts, including supermarkets, households, tea houses, hair salons, shopping malls, food markets, photographic studios, and others in Jishou from December 2012 to July 2021. The corpus comprises approximately 70 h of interaction by 97 participants, including 61 women and 36 men, all of whom are native speakers of the Jishou dialect. Their ages range from the mid-20s to the late 80s. Various contexts and combinations of participants are chosen to guarantee that the research findings are not context- and participant-specific.

The 70 h of interactions were transcribed using the *Pinyin* romanization system without tone marks, in accordance with the conventions of CA (Atkinson and Heritage, 1984), with slight modifications^5^.

Each transcribed Jishou dialectal turn is accompanied by two lines of an English translation. The first line provides a literal translation of each Jishou dialectal element, while the second line offers an idiomatic English equivalent, as is shown in excerpts 1–5.

### 3.3. The contextual distribution of the turn-medial particle *dai*

#### 3.3.1. The number and relative frequency in eight different contexts

As shown in Table 1, there is a noticeable discrepancy in *dai'*s distribution in eight different contexts. More specifically, out of a total of 2,167 instances in the present corpus, 1,851 instances occur overwhelmingly in supermarkets (1,080) and households (771) contexts, respectively. Other contexts with a dramatically lower frequency of *dai* are food markets (160), shopping malls (90), photographic studios (23), tea houses (18), hair salons (12), and others (13). A simple calculation reveals that the number of *dai* in supermarket and family contexts is about six times that of the total number of the other contexts. The data presented in the supermarkets are conversations among four sisters (M, A1, A2, and A3). In contrast, those in the households consist of family members (M, F, and R), demonstrating that the turn-medial particle *dai* is more likely to be used among participants with more intimate social relationships.

**Table 1:** The number and relative frequency of *dai* in eight different contexts.

**Contexts**	**Numbers**	**Relative frequency**
Supermarkets	1,080	55924^1^ : 1080 ≈ 52: 1
Households	771	44328: 771 ≈57: 1
Food markets	160	36554: 160 ≈ 228: 1
Shopping malls	90	36023: 90 ≈ 400: 1
Photographic studios	23	42066: 23 ≈ 1829: 1
Tea houses	18	42460: 18 ≈ 2359: 1
Hair salons	12	39088: 12 ≈ 3257: 1
Others	13	2241: 13 ≈ 172: 1
Total	2,167	298684: 2167 ≈ 137: 1

^1^This refers to the total number of Chinese characters in each context.

However, as shown in Table 1, although its frequency is lower, the occurrence of *dai* in seven other contexts involving participants with relatively more distant social relationships is not an isolated linguistic phenomenon. As will be shown later in this article, the speakers' negative stance for complaining and criticizing is displayed and partly achieved by the *dai* turn-at-talk. In addition, pursuing interpersonal harmony in interaction has been suggested to be the essence of human beings' rationality (Ran, 2012: p. 1); thus, in this study, there are fewer instances of face-threatening *dai* turns-at-talks in the seven other settings with participants in more distant social relationships, which is understandable.

#### 3.3.2. The relative frequency of *dai* in sequential positions

Sequential position refers to the relative position of adjacent turns within an adjacency pair consisting of two or more turns with conditional relevance in turn-taking (Schegloff, 1996). Previous studies (Schegloff, 1982; Couper-Kuhlen, 1996; Goodwin, 2000; Wu, 2004; Couper-Kuhlen and Selting, 2018) have found an “interaction” between particles' sequential positioning and their syntactic features, meanings, and functions. As Table 2 demonstrates, the turn-medial particle *dai* in an initiating turn, compared with that in a responsive turn, has a higher relative frequency. The fact that the sequential positioning influences the relative frequency of *dai* shows an interactive relationship between syntactic features, meanings, and functions of the turn-medial particle *dai* and its sequential placement in the interaction.

**Table 2:** The relative frequency in different sequential positions.

**Sequential position**	**Number and relative frequency**
Initiating turn	1,347
Responsive turn^1^	820
Total	2,167

^1^We have included 172 *dai* turns-at-talk in the third position in the responsive turns.

In this section, we have discussed the “interaction” between *dai* and its distribution in different contexts and sequential positions. Then, the interactional functions of the turn-medial particle *dai* from the perspective of CA will be described empirically.

## 4. Syntactic functions and prosodic features of the turn-medial particle *dai*

To date, there have been very few studies of *dai* in the Jishou dialect. This section will be dedicated to its syntactic functions and prosodic features.

### 4.1. Syntactic positions

Syntactically speaking, there are three main positions for the turn-medial particle *dai*: (1) being between the nominal subject and predicate, as is presented in the excerpts (6a, b); (2) following a sentence-initial adverbial, as is shown in excerpts (6c, d); (3) following a prepositioned object, as is presented in the excerpts (6e, f). Overall, the turn-medial particle *dai* is referred to as a marker dividing the sentential “theme-rheme.”

(6)     a.  *wo  **dai**   yi      yang     mei      de*.
               I     PRT one   C          N         gain
               I     DAI  have gained  nothing.
b.       *ta   sun    dou    hao   da    le*,
*         3sg   grandson    already  very    old    PRV,*
          *ni*   **dai**   *hai      mei you*.
*          you   PRT   still     N have*
His grandson has already grown up to be an
older child (but) you DAI do not have (your own) yet.
c.       *zhe ge      shihou **dai**   loufang   hai      jicongjicong.*
         this C       time     PRT  buildings still    everywhere
         In recent times     DAI, buildings exist   everywhere.
d.       *na     tian   lai    **dai**:*
*          that day   come     PRT*
*          wo    hai    yao    tamen    tuoxie   a.*
*          I     then   ask    them    take off       shoes    Q*
(Did it mean that) I should ask them to take off (their)
shoes on the day they came DAI (to my house)?
e.       *rou     dai            wo     de      ji           ke      chi.*
          meat   PRT         I         get    a few     C       eat
          I         (only) got a few dices of meat  DAI  to eat.
f.                                *anhao_anhao dai                mei*
                                                                              *kan dao*.
           cipher_cipher PRT                 N                  see ASP
           (I) have           not seen          the cipher     DAI (at all).

### 4.2. Sentential functions

The turn-medial particle *dai* in declarative sentences is shown in excerpts (7a, b ).

(7)      a. *ta   **dai**   gang,     zhe       shui       hui      leng*.
          3sg     PRT say,        this      water     will     cold
           It was him, DAI, who said the water would get colder.
b.        *dianshi     dai       ta     dou       mei  kan*.
           television PRT    3sg   already   N    watch
           He            hardly ever  watches TV   DAI.

In addition, the turn-medial particle *dai* can also be used in exclamatory sentences to express the speaker's assessment directly. As presented in excerpt 7c, *dai is* mainly used in responsive turns to evaluate a prior turn's statement.

(7)c (Supermarkets)

((A1 and M are making a comparison of transactions between two suppliers))

A1:     *na    ta     shengyi   mo          bu    hao     a?*
           then 3sg  business  Q             N    good   Q
           then isn’t  his          business  in    good   condition?
M:
           → *yo::    renjia      haoduo     dou      shi       zhijie     gei*
*           oh:: others many already   be    direct   give*
*           chaoshi    songhuo,    ta     dai:::*
*           supermarket deliver goods, 3sg PRT*
Oh::, many other suppliers directly deliver goods to
supermarkets (this is good business), but he (his business) DAI (is not good)

The turn-medial particle *dai* is completely incompatible with interrogative and imperative sentences based on the present corpus.

### 4.3. Prosodic manifestation

There are two types of prosodically different *dai*s in the present corpus; one of them, the unmarked *dai*, is produced with a flat, low pitch and demonstrates prosodic features closer to what has been described for particles in the literature, while the other, the marked *dai* (marking as *dai*^m^), does not. These marked tokens are produced either with a markedly high pitch or with some dynamic pitch movements, such as a rising or a falling-rising pitch contour. The unmarked *dai* is considered the main prosodic manifestation, as presented in excerpt 8:

(8)      a. *zhe ge       shihou   dai    loufang     hai      jicongjicong*.
           this  C          time      PRT   buildings  still     everywhere
           In     recent  times     DAI,  buildings  exist   everywhere.
b.        *rou   dai     wo   de     ji     ke             chi.*
           meat PRT   I      get    a     few           C    eat
           I       (only) got a few dices of meat   DAI to eat.

The connotation of a segment following the turn-medial particle *dai* can be inferred from the context so that speakers can omit the following segments. In this case, *dai* manifests itself as a marked one, as presented in excerpts 9a and 9b:

(9)a (Food Markets)

((S and D are coworkers in a food market. S grumbles about D's eating outside))

S:       *wo dengxia chuqu             chi            suanlafen.*
           I  later go outside              to eat        (local snack)
           I will go outside to eat hot and sour rice noodles later.
D:       → *wo fan dou       bang ni    zhu   le,     ni       **dai**^m^*::
            I        rice already help you cook PRV, you    PRT::
  I have already cooked the meal for you, but you DAI (do not eat it).
b (Shopping Malls)
    ((Z is B’s client. They complain about their dark circles to each other))
Z:        *ni            kan wo dou            you   heiyanquan*.
           you see,   I     already have dark circle
           Look,       I     already have dark circles.
B:       → *wo haiyao geng    hei      xie,  wo  **dai**^m^*::
           I          even   more     black   C,  I      PRT::
           Mine (dark circles) are even worse; my dark circles
           DAI (is so heavy).

## 5. Sequential functions of the turn-medial particle *dai*

This section presents findings from a detailed analysis of the negative stance marker *dai* in the initiative and responsive turns, respectively. The particle mainly contributes to achieving a negative stance through increasing variation in volume, pitch, stress, and other prosodic resources; different uses of gestures and other types of embodiment; and accounting or other types of orientations toward these turn-at-talks, etc.

### 5.1. Initiative turn

In the present corpus, *dai* occurs regularly in initiating turns where the speaker expresses a complaint or criticism toward a prominent piece of information in that turn, as is illustrated in excerpt 10:

(10) (Households)

(M first complains to F that A1's relatives ate up all the agents' consigned beverages, and then M and F reach an agreement that it is not good for people to drink too many beverages)

1M:     →   *yi jian **dai**::,    aiyou*.
*            one C PRT, (exclamation)*,
*            A2 shuo,    hai      mei    fanying    guolai*,
*            A2    say,   N        react   ASP*,
*            mei   you    le*.
*            N     have    PRV*.
            A full box DAI (of beverages) , well, A2 said, had been
            emptied before others knew it.
2M:     *A1 la,    ta    wu     naxie     qinqi    yi    lai,     jiu*
*            A1 PRT,   3sg   family:   once    those    relatives*
*            come*,
*            han      tamen   chi*.
*            ask   them       to    eat*.
            When A1’s relatives visit, she always treats them
            (with these beverages).
2F:       *A1  han, [<kuai    chi  kuai    chi*
            A1  say, [ <quick   eat quick   eat
            A1  told  them       to   drink    freely.
3M:      *um.   zhexie  chi       duo       le      bu      hao.*
             PRT,  these   eat      many    CRS   N       good.
             Um, it is        harmful to drink too  many beverages.
4M:      *Tamen shuo hanyou fuermalin, hai you naxie sesu.*
             they say contain formalin, then have those pigment
             They said that there are formalin and pigments in
             those beverages.
5F:        um,   you    dian,    duoshao    you    xie.
             PRT,    have C,     more or less have C
             True, there are, more or less.
6M:       *xiaode man, chi    naxie chi      duo     le        bu     hao     a.*
              know   Q,     eat    those eat      many   CRS   N      good   PRT
              Get       it?   It is not       good for you to drink too many (beverages).

In excerpt 10, the *dai* turn-at-talk delivers some prominent information about the conversation (line 1); that is, A1 complains that A1's relatives emptied a box full of beverages, and the verbal practice “*yi jian* ***dai::/***a box full DAI (of beverages)” produced with a prolonged *dai* invites other speakers to join in with speaker M's complaints and criticisms. Preceding the *dai* turn, speaker M's complaints and criticisms of A1's selfishness are evident throughout the conversation. Primarily, through the choice of words conveying a sense of complaining (*naxie/*those*, buhao/*not good), speaker M expresses a recognizable negative stance. In line 1, M showed her discontent by referring to A1's relatives with the deictic expression “*naxie*” (those). According to Fang (2002), the deictic word “*na*” (that) indicates that the stated event lies in the marginal area of a speaker's inner world and is used to express a negative or disapproving stance toward the event. The *dai* turn-at-talk simultaneously elicits F's evaluation of A1's selfish behavior: given that the practice of giving an identical repetition of A1's utterance “*kuaichikuaichi*” (eat at a quick pace) in a quick tempo, F vividly depicts the situation where A1 urges her relatives to drink free beverages, implying his complaints on such selfish behavior. Moreover, in lines 4–6, both M and F believe that A1's behavior is not only selfish but harmful to her relatives' health. Lexical and prosodic resources form the evidence of the conversation as being “affect-laden”; reflexively, the *dai* utterance itself shapes the development of the interaction; that is, as the whole conversation seems to be complaining about the event from the start with the *dai* turn-at-talk, the negative stance is co-constructed in the whole conversation.

Excerpt 11 provides another instance in which the *dai* turn-at-talk is used to express complaints and criticism toward a recipient. *dai* turn-at-talk, in this case, mainly exists among family members:

(11) (Supermarkets)

((M rebukes R for his improper wearing of shoes in the hot summer))

1M:       ni    dai     chuan   qi     na       xiezi        huilai,
              you PRT   wear   CRS that     shoes        back,
                               zheme re.
                               so        hot.
             How could you     DAI wear these shoes back in
                                                                               this heat?
2R: *       ai::ya::,  chuan shenme xiezi? ni xiaode shenme?*
              (exclamation), wear what shoes? you know what
              Ah, what’s wrong with my shoes? You know
              nothing (about fashion)

As shown in excerpt 11, M uses *dai* turn-at-talk to blame R for wearing high heels in the hot summer. Then, R responds to the previous turn with the stressed exclamation “*aiya*”(Ah) and two consecutive rhetorical questions, expressing his strong impatience. R's responses indicate that he has M's complaints in the previous turn. Considering this, through the selection of “*na*”(that) as in excerpt 10 and syntactic design—rhetorical questions—speaker M showed her negative stance, and the whole conversation is filled with a negative flavor, such as the prolonged injection *aiya*.

Despite *dai* turns-at-talk in the data signaling speaker's negative stance, the above excerpts demonstrate that the particle *dai* itself can be used to construct complaints and criticisms to some extent. Excerpt 12 is a case in point:

(12) (Supermarkets)

((M, A2, A3 gossip about their relative—CQ. They thought that CQ bought PH a hat because CJ (PH's mother) buys almost all the goods that she needs in CQ's shop.))

1A2:      *zhe maozi hao chou*.
              this hat very ugly
             This hat is really ugly.
2M:       *xiao yaer bu guan*.
              little boy N care
              Little boy does not care about that (he wears an ugly hat).
3A2:      → *ta ((CQ)) dai hao, ta gei ni yaer mai maozi la*.
              3sg PRT good, 3sg give your child buy hat PRT
              She DAI (is) not bad, she bought your son this hat.
4A3:      *CJ changsi dao ta((CQ)) nail mai dongxi da*=
              CJ often go 3SG there buy thing PRT=
              CJ often goes to her((CQ)) shop to buy something=
5M:        =*ta ((CJ)) zhaogu ta((CQ)) shengyi da*.
               =3sg patronize 3sg business PRT
               She ((CJ)) patronizes her ((CQ)) place.
6A3:      *zhe yaer ((CJ)) shenme dou dao ta((CQ)) naer mai*,
              this guy what all go 3sg there buy
              This guy ((CJ)) goes to her ((CQ)) shop
              whenever she needs to buy something.
7A3:      *ni bu gei ta mai, ta bu gei ni yaer mai*.
              you N give 3sg buy, 3sg N give your child buy
              (if) you do not buy something
              from her ((CQ)) shop, she will
              not buy your child (the hat).
8A3:      *buguo ta((CQ)) ye keyi, ni de ge maozi dai. hhh*
              but 3sg also okay, you get C hat wear(laugh)
              But she ((CQ)) is not bad, you got a hat from her (at least). hhh
9A2:       *jiu shi jiang lo, gei ni mai maozi*.
               then be say PRT, give you buy hat
              That’s right. She ((CQ)) bought your (son) a hat.

In excerpt 12, “ta *dai* hao” (she is not bad) is a format of “*dai* + good.” The particle *dai* contributes to the display of a negative stance to some extent, whereas the adjective “hao (good)” usually displays a positive stance. Thus, combining these two words is supposed to accomplish a speaker's mixed stance; that is, the topic in focus is both bad and good. Excerpt 12, involving positive and negative aspects simultaneously on a topic in a conversation, might serve as evidence regarding *dai* itself as a negative stance marker to some extent. To begin with, in line 1, A2 is saying, “*zhe maozi hao chou* (This hat is really ugly),” thereby displaying a strong negative stance toward the hat's style and design. Then, M's initiation, “*xiao yaer buguan* (Little boy does not care about that he wears an ugly hat)” in line 2, elicits A2's response, “*ta ((CQ)) dai hao, ta gei ni yaer mai maozi l a*(She is not bad, she bought your son this hat.)”. At first, A2 complains that the hat is ugly. M seems to criticize that complaint by claiming that the boy does not care about being ugly, thereby also implicitly defending the giver, and A3 seems to take up on the implicit defense of the giver by explicitly praising the giver for giving the hat. A3's lines 4 and 6 provide evidence for A2's implication: A3 thinks that it is because CJ often takes care of CQ's business that CQ buys a hat for PH, which indicates that this action should not be CQ's willingness but a kind of exchange of benefits. In addition, A2 uses the intensified responsive marker “*jiushi jianglo* (That's right)” in the closing line 9 to show that she does not just agree with A3 but rather positively assesses that A3 repeated her own previous *dai*-complaint in line 3 (Qu, 2006: p. 78; Yao, 2012: p. 76). From the above, we can conclude that “*ta dai hao*” (she is not bad) is a mixed stance toward her (CQ), where the positive aspect comes from the adjective “*hao* (good)” and the negative aspect, without any doubt, is brought about by the turn-medial particle *dai*.

### 5.2. Responsive turn

A speaker mainly uses the turn-medial particle *dai* in responsive turns to construct a self-accusation when faced with criticisms from others. We count 820 *dai*s in the responsive turn and find that 648 cases, or about 80% of the total, belong to this pattern, as is illustrated in excerpt 13:

(13) (Hair Salons)

((T questions hair stylist H for ignoring his regular customer C10))

1T:          *ta    han   ni    ji    sheng*,
*               3sg call you several sound,*
*               wo kan ni dou mei li ta.*
*               I see you even N respond 3sg*
               She called you several times, but I saw that you did not
               respond to her.
2H:         → *wo **dai**^m^ zhe tiao yanjing bu ren ren. hhhh Qishi*
               *man, dou shi shuren*.
               I PRT this C eyes N know person (laugh). Actually PRT, all
               be acquaintances
               I’m DAI not good at recognizing others. hhhh. Actually we
               are all acquaintances.
3T:          *oh, deng xia gei renjia jieshi yi xia*,
               PRT, wait C give people explain one C,
               Yeah, then you’d better explain to her,
4T:          *yaoburan jiang ni bu li ren hhhh*
               otherwise say you N respond people (T and H laugh together)
               otherwise, she would think that you ignore her
              (deliberately). hhhh

In excerpt 13, in the face of T's blame for “ignoring the regular customer,” H uses a format of “*wo dai*^*m*^(I *dai*) + reason” to express self-accusation in a self-deprecating way. In excerpt 14, J, in the third position in the responsive turns, responds to the blame for “mistaking soy sauce for vinegar” from T and R in the same way.

(14) (Tea Houses)

((T, R and J are close friends. T and R blame J for his mistaking soy sauce for vinegar))

1T:         *aiya, ni gao shenme la? Shi jiangyou, ni kan qingchu qi lo*.
              (exclamation), you do what PRT? Be soy sauce, you
              see clear ASP PRT
              Ah, what are you doing? It is soy sauce (not vinegar),
              you had better see more clearly (next time).
2R:        *na tiao J ou*.
              That C J PRT.
              What a (sloppy) person J is.
3J:          → *wo **dai**^m^ dou meng le, wo yiwei zhe tiao shi cu*.
               I PRT even stupid PRV, I think this C be vinegar
               I DAI was thoroughly lost, I thought it was vinegar.
4:            *hhhh*.
               (J, T and R laugh)

What is notable in the above two excerpts is that their self-deprecating blame on themselves triggers the “laughter” of participants. The laughter, caused by H's neglect of a regular customer and J's ridiculous mistakes, is employed by the speakers simultaneously to alleviate the embarrassment caused by the criticism (Levinson, 1983: p. 70). In other words, the non-verbal practice of “laughter” demonstrates that other participants accurately catch the self-deprecating blame of the speaker's turn.

The turn-medial *dai* is also used by speakers to directly express negative evaluations of the participants in the interaction. Nevertheless, in line with the politeness principle, such direct complaints and criticism are not common. This conclusion is supported by statistics, which indicate that *dai* of this type occurs 57 times, accounting for only a small portion (7%) of the entire corpus of 820 *dai*s in responsive turns, as presented in excerpt 15:

(15) (Photographic Studios)

(( A customer complained that studio employee S accidentally deleted a photographic plate. S' boss is now talking to him about it.))

1S:          *wo gang gen ta jieshi le, ta ziji jiang bu yaojin, ta*
*               diannao litou cun de you*.
               I just with 3sg explain CRS, 3sg self say N matter,
               3sg computer in save CSC have
               I just explained it to him, and he said that it did not
               matter, for he had already saved them (the pictures) on
               his computer.
2Q:         → *ni **dai**^m^* zuo shi tai meiyou zhuntou le, renjia tousu
                ni le, ni hai guai renjia.
                You PRT do a thing so N reliability PRT, other
                complain you PRT, you still blame other
                You DAI are so unreliable. The customer has
                complained about you, and you are still blaming
                the customer.
3S:           *na zhende buhaoyisi, wo buxiaoxin shan cuo de*.
                Then really sorry, I careless deleted mistake ASP
                I’m really sorry, then. I deleted (the pictures) by accident.

In excerpt 15, speaker Q's negative stance in line 2 can also be verified from the next turn by recipient S: S uses an apologetic expression “*buhaoyisi* (I'm sorry)” with an intensifier “*zhende* (really)” to respond to Q's criticism in the prior marked *dai* turn, displaying that he orients to *dai* turn-at-talk as a negative stance-laden statement.

## 6. Conclusions

Particles are viewed as key contributions to speakers' fluency, although some are stigmatized as informal, disfluent elements of speech (Crible et al., 2017; Degand, 2018; Degand and Van Bergen, 2018). By combining syntax, pragmatic functions, and syntagmatic variables (co-occurrence and clusters of words, pauses, silence, etc.), as well as rich corpus-based observations, our study might obtain a more comprehensive picture of the turn-medial particle *dai*, which is an overt marker of speakers' negative stance, including complaining and criticizing. The study shows that a negative stance is treated as an emergent product shaped continuously by diverse factors in talk-in-interaction and shapes the subsequent development of conversation. In other words, CA of the turn-medial particle *dai* takes an interactional approach, which is different from some previous studies of stance (e.g., Biber and Finegan, 1988, 1989; Field, 1997), whose focus has been on the realization of linguistic stance markers. Specifically, the current study is focused on how stance can be accomplished in interaction by means of linguistic and other resources, such as context, sequential positioning, and prosodic manifestation.

Context is a key factor in accomplishing language functions (Hopper, 1998; Heine and Kuteva, 2002). In this study, displaying the negative stance is both “context-sensitive” and “context-renewing.” On the one hand, the turn-medial particle *dai* exists in contexts featuring complaints and criticisms with high frequency, which further embodies and reinforces its interactional function by absorbing the sense of the complaints and criticisms implied in the context. On the other hand, there is a “reflexive relationship” between the particle *dai* and the context in which it occurs. The speaker uses it to establish a “stance frame” and sets a baseline of negative evaluation for the subsequent conversation.

Furthermore, the turn-medial particle *dai* interacts with different sequential positions and realizes multivariate interactional functions. Positioning the particle in the first pair of an adjacency pair allows the speaker to initiate a new sequence and take control of the interaction. Thus, a turn with *dai* in this sequential position is responsible for drawing the listener's attention to key information in the conversation. When the particle *dai* is positioned in the second or third part of an adjacency pair, it refers to the current speaker's response and achieves a negative assessment of what others just said or intended in a preceding turn (or turns).

With respect to the prosodic manifestation of the turn-medial particle *dai*, we noted that there are two types of distinctive *dai* in the present corpus: the unmarked *dai* produced with a flat, low pitch, and the marked *dai* produced either with a markedly high pitch or with some dynamic pitch movements, such as a rising or a falling-rising pitch contour. Unmarked forms occur regularly in initiating turns where speakers complain or criticize a prominent piece of information. Unmarked *dai* registers the matter being addressed as new information. A speaker mainly uses the unmarked *dai* in responsive turns to construct a self-accusation when faced with criticism from others.

However, the marked *dai* is characteristically used to register a stronger negative stance. It is commonly used to alert the recipient to some negatively valenced interactional work that the *dai*-suffixed turn-at-talk is attempting to accomplish. It is worth noting that the connotation of a segment following a marked *dai* can be inferred from the context so that speakers can omit the following segment; that is, the *dai* utterance is prosodically, syntactically, and pragmatically excluded.

The examples in “Section 5” reveal that both *dai* in initiating turns and in responding turns are indexing a complaint or criticism already established in the sequence. *Dai* also serves different functions in the first, second, or third positions, whether in a turn of informing or in a turn that both receives and informs. In other words, while unmarked *dai* in the first pair of an adjacency pair and marked *dai* in the second or third position can be used to register a new delivery as well as a new assessment, the former characteristically invokes a sense of emphasis, which is not displayed in the latter, and the additional layer of import exhibited in the use of marked dai may be achieved by the interaction among distinctive prosody and sequential position.

Our findings regarding the interactional functions of the turn-medial particle *dai*, with its distinctive prosodic characteristics, contribute to our existing body of knowledge on the Jishou dialect. The present study, based on an extensive corpus of naturally occurring interactions, is a new attempt to describe and explain the interactional functions of the turn-medial particle *dai* and the underlying logic and regularity using the CA. The work done here is just the tip of the iceberg; it serves only as a starting point for more investigation. Further CA research is needed to develop the interaction analysis of a wide range of discourse particles in the Jishou dialect from the following three aspects: (1) investigating the actual usage of particles based on naturally occurring interactions; (2) implementing positionally sensitive grammar by carrying out a dynamic analysis of different particles' sequential organization; and (3) exploring the patterns and emergent conditions of different particles. Apart from occurring in both initiative and responsive turns, there seems to be a pattern in the use of the particle *dai* in the current turn, e.g., X *dai*, + clause, X *dai* + the rest of the clause, X *dai*^m^:::. Its role in these structures may have been different, such as a topic marker for the division of theme and rhyme. As an important discourse marker, the analysis of what particle *dai* contains should not be limited to the sequential position but its position in the turns. More research is also needed to elucidate this matter.

## Data availability statement

The original contributions presented in the study are included in the article/Supplementary material, further inquiries can be directed to the corresponding author.

## Ethics statement

Ethical review and approval was not required for the study involving human participants in accordance with the local legislation and institutional requirements. Written informed consent to participate in this study was not required from the participants in accordance with the national legislation and the institutional requirements.

## Author contributions

FL contributed for article writing. XL and RL for data collection and analysis. JZ for data collection. All authors contributed to the article and approved the submitted version.

## Footnotes

^1^It should be noted, however, that the term “particle” is occasionally used as a term of convenience as a substitute for the term “turn-medial particle” in this study.

^2^“Negative interactional contexts” refer to situations in which speakers express their negative attitudes, potentially challenging the listener's social image, such as through complaints, objections, refusals, warnings, and so on. For example, Wu's (2004) study of final particles in Mandarin Chinese using Conversation Analysis has demonstrated that the particle *ou* at the end of a turn serves as a warning to the listener of negatively-valenced interactions such as complaints, disagreements, warnings, jokes, declines of requests, rejections of expectations, or denials of presuppositions.

^3^The main participants' coding is found in Appendix I.

^4^We have obtained informed consent from the participants in the recordings done in public places.

^5^The transcription and glossing conventions employed in the excerpts are provided in Appendices II and III.
