# Supplementary material for: Linguistic expressions of negative stances: A conversation analysis of turn-medial particle dai in Jishou dialect (Hunan Province, China)
Source: Front Psychol. 2023 Feb 21;14:1018648. doi: 10.3389/fpsyg.2023.1018648 (PMC9989467; doi:10.3389/fpsyg.2023.1018648)
Supplement: Supplementary file 1 [file Data_Sheet_1.pdf]

Appendix I: the main participants' coding

M	=	first author's mother
F	=	first author's father
R	=	the first author
A1, A2, A3	=	salesman #1, #2, #3

Appendix II: Transcription conventions

[	A left bracket denotes the point at which the talk of another speaker, which appears on the following line attributed to another speaker, overlaps the current speaker's utterance..
=	Equal signs are used to mark that there is no interval between adjacent utterances by different speakers, with the second being followed by the first.
::	Colons are used to indicate the prolongation or stretching of the sound just preceding them. The more colons, the longer the stretching.
｡｡	Double-degree signs indicate that the talk between them is quieter than the surrounding talk.
_	A hyphen after a word or part of a word indicates a cut-off or a self-interruption.
<	A left-facing arrow marks the onset of a stretch of talk that is markedly rushed or compressed.
→	An arrow sign of this type specifies the target of focus in the transcription.
* *	Asterisks are used to indicate that the utterance between them was followed by laughter.
(( ))	A double parenthesis is used to mark the transcriber’s descriptions of events.
( )	A parentheses is used to mark the speaker’s implication or extra explanations.

Appendix III: Glossing conventions

ASP	aspectual marker
BA	the *ba* marker in the *ba* construction
CRS	currently relevant state (*le*)
CSC	complex stative construction
C	classifier
N	negator
PRT	particle
PRV	perfective (-*le*)
Q	question marker
3sg	third person singular pronoun
